# Isolation and characterization of extracellular vesicles and future directions in diagnosis and therapy

**DOI:** 10.1002/wnan.1835

**Published:** 2022-07-27

**Authors:** Karina P. De Sousa, Izadora Rossi, Mahamed Abdullahi, Marcel Ivan Ramirez, Dan Stratton, Jameel Malhador Inal

**Affiliations:** ^1^ Bioscience Research Group, School of Life and Medical Sciences University of Hertfordshire Hertfordshire UK; ^2^ School of Human Sciences London Metropolitan University London UK; ^3^ Federal University of Paraná Curitiba Brazil; ^4^ Carlos Chagas Institute (ICC) Curitiba Brazil; ^5^ Open University The School of Life, Health and Chemical Sciences Milton Keynes UK

**Keywords:** EV analysis, EV characterization, EV therapeutics, extracellular vesicles, isolation methods

## Abstract

Extracellular vesicles (EVs) are a unique and heterogeneous class of lipid bilayer nanoparticles secreted by most cells. EVs are regarded as important mediators of intercellular communication in both prokaryotic and eukaryotic cells due to their ability to transfer proteins, lipids and nucleic acids to recipient cells. In addition to their physiological role, EVs are recognized as modulators in pathological processes such as cancer, infectious diseases, and neurodegenerative disorders, providing new potential targets for diagnosis and therapeutic intervention. For a complete understanding of EVs as a universal cellular biological system and its translational applications, optimal techniques for their isolation and characterization are required. Here, we review recent progress in those techniques, from isolation methods to characterization techniques. With interest in therapeutic applications of EVs growing, we address fundamental points of EV‐related cell biology, such as cellular uptake mechanisms and their biodistribution in tissues as well as challenges to their application as drug carriers or biomarkers for less invasive diagnosis or as immunogens.

This article is categorized under:

Diagnostic Tools > Biosensing
Therapeutic Approaches and Drug Discovery > Nanomedicine for Oncologic Disease
Therapeutic Approaches and Drug Discovery > Nanomedicine for Infectious Disease

## Graphical abstract

Fundamental research on EV isolation, standardization and characterisation will bring about translational medical diagnostics and therapy.

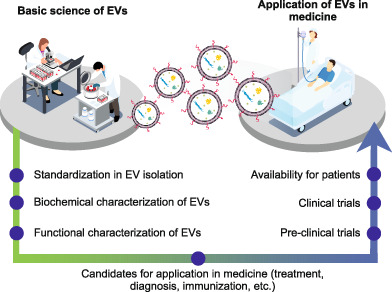

## 1 INTRODUCTION

Extracellular vesicles (EVs) are lipid bilayer vesicles released in an evolutionary conserved manner by cells, from prokaryotes to higher eukaryotes and plants (Stotz et al., 2022; Yáñez‐Mó et al., 2015). This review will focus on exosomes and microvesicles (MVs), two subgroups of EVs, as therapeutic applications of apoptotic bodies (large EVs [lEVs]) are still in their infancy (T. K. Phan et al., 2020). The biogenesis of exosomes involves endocytosis and formation of multivesicular bodies (MVBs) containing intraluminal vesicles (ILVs) formed by the internal budding of the endosomal membrane. ILVs can be degraded by fusion of MVBs with lysosomes or secreted into the extracellular space by fusion of MVBs with the plasma membrane (PM). From then on, these vesicles are considered “exosomes,” having a diameter that varies from 30 to 200 nm. The protein topology in exosomes remains the same as in the PM of the releasing cell. On the other hand, MVs are released directly into the extracellular matrix through the external budding of the PM and are heterogeneous in size, ranging from 100 nm to 1 μm (Kalra et al., 2016; Van Niel et al., 2018). Biogenesis pathways for budding MVs are still being elucidated (Catalano & O'Driscoll, 2019; Kholia et al., 2015; Sedgwick & D'Souza‐Schorey, 2018). The overlap of physical characteristics (such as size) between MVs and exosomes, added to the lack of specific markers that differentiate them, has made it challenging to study these two populations individually. Therefore, the International Society for Extracellular Vesicles (ISEV) advocates the use of the generic term “extracellular vesicle” (EV), unless authors can establish specific markers of subcellular origin that are reliable in their experimental models or can name them based on operational terms for the EV subtypes they refer to, such as the use of lEVs (which would mostly correspond to MVs) and small EVs (sEVs, mostly exosomes).

The first function attributed to exosomes following their discovery (Harding et al., 1983; Pan et al., 1985) was the elimination of unwanted cellular proteins (Johnstone et al., 1987). Currently, exosomes and MVs are implicated with a variety of biological processes, including tissue remodeling, transport of intercellular material, metabolic regulation, protein removal and trafficking, among others (Iraci et al., 2016). Although their physiological role in homeostasis is recognized, the main interest has been to investigate EV participation in pathological conditions, such as cancer (M. P. Bebelman et al., 2018), autoimmune diseases (Antwi‐Baffour et al., 2010; J. Tian et al., 2020), infections (Antwi‐Baffour et al., 2019; Caobi et al., 2020; Cestari et al., 2012; De Sousa et al., 2022; Evans‐Osses et al., 2017; Joffe et al., 2016; Rodrigues et al., 2018; Rossi et al., 2021), and neurodegenerative disorders (Lange et al., 2017; Xiao et al., 2021).

These functions are performed by transferring encoded information and bioactive molecules in the form of cytokines, lipids, genetic material, proteins, peptides, and other macromolecular elements to recipient cells (van Niel et al., 2018). EVs are stable structures, their contents being protected from degradation processes. They are easily taken up by many cell types and can act locally or circulate through various body fluids, including blood and lymph, resulting in a systemic response (Mathieu et al., 2019). They have been shown to display homing capacity (Cesi et al., 2016), are able to cross the blood–brain barrier (Morad et al., 2019), and are nonimmunogenic (Cestari et al., 2012; Villa et al., 2019). These characteristics make EVs very attractive acellular, biocompatible agents for drug delivery, immune mediation, cancer therapy and even for regenerative medicine (Fais et al., 2016). Furthermore, EVs may provide a means for minimally invasive diagnostics and therapeutics. Attesting to the interest in these vesicles, there are currently a number of studies appraising their various clinical uses (C. Choi, 2022; T. H. Phan et al., 2022).

## 2 METHODS FOR THE ISOLATION OF EVs

EVs can be studied both in vitro and in vivo, and are found in many biofluids (blood, milk, saliva, urine, amniotic fluid, semen, cerebrospinal fluid, ascites) (Bano et al., 2021), demonstrating their role in cellular communication between distant body compartments.

The specific method used to isolate EVs greatly influences the yield and purity of isolated samples. Numerically, it is generally accepted that freshly acquired and processed samples yield higher numbers of vesicles, but this is not always possible. In addition, EV isolation is complicated not only by their nanosize but also by contaminants which may be co‐isolated with EVs (including cellular debris and interfering components: lipoproteins, protein complexes, aggregates) (Ramirez et al., 2018). Importantly, it has been shown that different isolation methods greatly impact the downstream analyses of EV cargo and physicochemical properties (S. Sharma et al., 2020; van Deun et al., 2014). The scientific community, mainly led by ISEV, has made efforts to standardize good practices for obtaining and characterizing EVs (Lötvall et al., 2014; Théry et al., 2018).

### 2.1 Ultracentrifugation‐based methods for EV isolation

Historically, ultracentrifugation protocols are the most widely used methods for EV isolation, both from cell culture media and body fluids. In fact, it is estimated that this method accounts for 81% of all EV isolations (Gardiner et al., 2016), as ultracentrifugation requires very little technical expertise, is an affordable technique over time and obviates the need for expensive and mechanistically unclear commercial EV isolation kits (X. Zhang et al., 2020). The process separates EVs from the other materials present in the sample based on their volume and physical properties using differential sequential centrifugation cycles at 4°C with forces of up to 120,000*g* applied directly to samples, there being little or no need for pretreatment of samples. Isolated EVs can be stored at 4°C until further analysis and should be used as soon as possible thereafter. Looking forward, EV analysis directly from samples, without the need to isolate them first, is an obvious goal.

Simple differential ultracentrifugation is a suitable method for concentrating EVs, but improvements to the technique have been introduced in order to obtain preparations of higher purity. In particular, buoyant density centrifugation methods (also known as isopycnic separation and zone centrifugation) adjusted to the specific density of EVs (1.13–1.19 g/ml) are often used to separate them from potential co‐isolated contaminants (Szatanek et al., 2015). This method yields single EV banding at their characteristic density zone, making collection of the vesicles simple. To achieve separation based on EV density (rather than weight), either a cushion or a gradient method can be used, reportedly with little effect on the number of vesicles collected (Yamashita et al., 2016). For both methods, solutions of sucrose or iodixanol are the most commonly used, but the use of iodixanol offers several advantages over sucrose solutions. Iodixanol is less viscous and thus easier to handle, metabolically inert and non‐toxic to cells and due to its lower osmolality is a better preservative of EV integrity and functionality ( K. Li, Wong, et al., 2018).

In comparison to ultracentrifugation, density gradient centrifugation has been shown to produce preparations with higher purity and yield (Duong et al., 2019; van Deun et al., 2014); however, this method is labor‐intensive, time‐consuming, and not suitable for high‐throughput applications. Furthermore, the method is more suitable for large sample volumes than for the processing of clinical samples. Importantly, it has also been observed that EVs isolated by ultracentrifugation show impaired functionality (Mol et al., 2017) or form aggregates (Linares et al., 2015), potentially linked to the damaging forces exerted on the vesicles during centrifugation at high speed.

### 2.2 Size exclusion‐based EV isolation

EVs can be isolated according to size and one of the most popular methods by which to do this is ultrafiltration (also termed microfiltration). This technique employs simple membrane filters with specific size exclusion limits (using pore diameters of 0.1, 0.22, or 0.45 μm in general), through which EVs in suspension are filtered (Grant et al., 2011) or in combination with other EV isolation methodologies (Stam et al., 2021), providing a fast and inexpensive method for separating EVs from bigger elements (Konoshenko et al., 2018). In our opinion, the term ultrafiltration should be confined to use of pore sizes below 0.1 μm, which are more commonly used to remove viruses. The EV preparations resulting from ultrafiltration consist of free‐standing single particles, not aggregates, which is favorable for downstream analysis. Additionally, ultrafiltration greatly reduces the likelihood of rupturing EVs, since the vesicles are not subject to the same forces and pressure required by ultracentrifugation methods; this likely explains why ultrafiltration recovers significantly more EVs than ultracentrifugation (Grant et al., 2011; Lobb et al., 2015; Yu et al., 2018). However, EV preparations obtained by ultrafiltration are often contaminated with molecules of a diameter similar to that of EVs, rendering the method less adequate for downstream proteomic analysis if used alone (Inal et al., 2013). To counter this, and due to the ease of combining ultrafiltration with ultracentrifugation, these two methods are often used together (Parimon et al., 2018; Y. Xu, Qin, et al., 2017).

Hydrostatic filtration dialysis techniques have likewise been used for processing larger sample volumes, as an alternative to direct ultrafiltration. This method has been shown to produce preparations that are enriched in EVs by up to 100 times when compared to ultracentrifugation‐based techniques and is less labor‐intensive and cheaper (Musante et al., 2014, 2017; R. Xu, Simpson, & Greening, 2017).

Size‐exclusion chromatography is becoming increasingly popular to isolate EVs by fractionation, resulting in preparations of high purity (Benedikter et al., 2017; Lozano‐Ramos et al., 2015). With this method, samples are filtered through a porous stationary phase; sample components with small hydrodynamic radii are able to pass faster through the pores and are thus eluted quickly, while those components with larger radii are excluded from entering the pores. Again, this method has been used in combination with ultracentrifugation (Onódi et al., 2018; Rood et al., 2010) and/or ultrafiltration (Benedikter et al., 2017; Nordin et al., 2015), with the added benefit of having a relatively low cost and short isolation time. Although generally accepted as a good method for EV isolation, concerns related to vesicle deformation and rupture have been raised, but these problems can be minimized by selecting the appropriate stationary fractionation column and the use of gravity alone to perform the chromatography separation. Accordingly, it has been shown that size‐exclusion chromatography can produce a high yield isolation while preserving biophysical and functional properties of the isolated vesicles (Foers et al., 2018; Gámez‐Valero et al., 2016; Hirschberg et al., 2021; Mol et al., 2017).

Asymmetrical flow field‐flow fractionation (AF4) systems may represent an improvement to size‐exclusion chromatography. While both methods work on the principle of size exclusion, the AF4 technique makes a parabolic flow run along a porous rectangular axis channel, carrying the sample and distributing particulate components based on their diffusivity: smaller particles diffuse further and are eluted earlier than larger ones (B. Wu et al., 2020). Successful EV isolations were obtained with this technique showing that it requires a smaller volume of starting material than in conventional chromatography and is able to produce EV preparations of high purity (Kang et al., 2008).

### 2.3 EV isolation by precipitation

Another alternative to ultracentrifugation is the use of precipitation methods. This technique, allows vesicle aggregates to be easily formed upon the addition to the sample of water‐excluding polymers such as polyethylene glycol (PEG) (García‐Romero et al., 2019; Weng et al., 2016) or lectins (Samsonov et al., 2016; Shtam et al., 2017) which force less soluble components out of solution allowing them to be subsequently precipitated by low‐speed centrifugation (Deregibus et al., 2016; Niu et al., 2017; Serrano‐Pertierra et al., 2019). Alternatively, the protein organic solvent precipitation (PROSPR) method (Gallart‐Palau et al., 2015), and the commercially available Total Exosome Isolation Reagent (ThermoFisher Scientific) have been proposed as inexpensive and quick protocols for EV isolation. This approach uses organic solvents to remove unwanted soluble proteins from complex biological fluids such as plasma, leaving behind a supernatant enriched in double‐membrane vesicles in suspension; the vesicles can easily be collected and separated by centrifugation. This approach reportedly produces high‐purity vesicle preparations suitable for downstream proteomic analysis, as serum albumin and other highly abundant plasma proteins are removed with the hydrophilic phase (Gallart‐Palau et al., 2015). The precipitation of EVs in culture supernatants with sodium acetate has also been shown to be a simple and inexpensive method. When isolated by this “salting‐out” technique, EVs are reportedly indistinguishable from those purified by ultracentrifugation (Brownlee et al., 2014).

Precipitation is an inexpensive method for EV isolation, with the added advantages of being easy to use, not requiring any specialized equipment, and being scalable for large sample sizes (Ludwig et al., 2018). However, the likelihood of co‐precipitating non‐EV material (such as protein aggregates, polymeric materials, other vesicles, or lipoparticles) is high. For this reason, several currently available commercial kits which rely on this method for EV isolation have introduced preisolation and postisolation steps aimed at minimizing contamination with subcellular particles and polymeric materials. Nevertheless, there are conflicting studies suggesting that those co‐purified molecules may not have a negative impact on the functional properties of isolated EV samples (Ludwig et al., 2018) and others suggesting that precipitation methods may be particularly detrimental to the biological activity of EVs (Baranyai et al., 2015), especially considering the potential cytotoxic effect and reduced viability observed in some cell lines following treatment with vesicles isolated using PEG and/or PROSPR precipitation (Gámez‐Valero et al., 2016).

### 2.4 Immunoaffinity capture‐based EV isolation methods

Although a rigorous definition of specific EV markers remains unclear, specific immunoaffinity techniques have been developed to take advantage of the presence of certain surface proteins and receptors (Brambilla et al., 2021; Brett et al., 2017; Ostenfeld et al., 2016; J. M. Wang et al., 2021). These techniques can easily complement other isolation methods, while offering increased efficiency, specificity and integrity in the recovery of EVs from complex and viscous fluids (Zarovni et al., 2015). Moreover, immunoaffinity methods are easy and fast to execute, and compatible with routine laboratory equipment. Immunoaffinity capture assays may, however, be negatively affected by antibody availability and the presence of these markers in the whole population (Gandham et al., 2020; Greening et al., 2015). Ideally, biomarkers should be solely or mainly expressed on the surface of the EVs and should also be fully membrane‐bound (without soluble variants) (P. Li et al., 2017). It may also be necessary to have a combination of markers to increase the chance of isolating a specific subpopulation of EVs.

Immunoaffinity assays aimed at isolating EVs can employ submicron‐sized antibody‐coated magnetic beads to increase the specificity, sensitivity, and yield of the isolation. This improvement is a consequence of the larger surface area, no sample volume limitations, and a near‐homogeneous capturing process (S. Chen, Shiesh, et al., 2020; Z. Chen, Yang, et al., 2020; Liangsupree et al., 2021; Zarovni et al., 2015). To apply this method of isolation, the immuno‐magnetic beads are first coated with antibodies against the EV‐associated surface molecules; next, they are incubated with the sample, forming EV‐magnetic bead complexes. Finally, the application of a magnetic field induces the movement of the complexes and separates them from the sample (Liangsupree et al., 2021). With this strategy, Zarovni et al. (2015) improved the recovery rate of vesicles from plasma samples by 10‐ to 15‐fold, when compared to ultracentrifugation. Another study combined ultracentrifugation, ultrafiltration, and magnetic immunoaffinity capture to isolate EVs, resulting in a high‐yield homogenous population of EVs that subsequently underwent a successful proteomic analysis (Mathivanan et al., 2010).

This approach therefore has obvious advantages: it is specific to the point of extracting subpopulations of EVs based on the expression of target markers; it ensures the integrity of the extracted EVs irrespective of vesicle size; it is relatively easy and quick to perform. However, it has been argued that this method cannot be applied to all sample types or all downstream analyses, as: (a) it is difficult to elute the EVs from the magnetic beads and (b) the non‐neutral pH and nonphysiological salt concentrations applied by this method may affect the biological activity of the EVs. A solution to the problem of eluting the EVs from the magnetic beads has been proposed (Nakai et al., 2016; Yoshida et al., 2017), by exploring the specific interaction between Tim4, an EV binding molecule, and phosphatidylserine molecules naturally present on the surface of EVs. The binding of these two molecules is Ca^2+^‐dependent, and intact EVs can easily be detached from Tim4 with the addition of Ca^2+^ chelators to yield high purity preparations. The authors have furthermore suggested that this approach can be adapted to ELISA and flow cytometry assays (Nakai et al., 2016). To further enhance the capacity of immunoaffinity assays for the isolation of EVs, several groups are developing exciting and comprehensive methods that couple magnetic immunocapture with mass spectrometry (MS; Ueda et al., 2014), multiplex bead‐based platforms (Koliha et al., 2016), on‐chip devices (Kang et al., 2020), nanowires (Dong et al., 2019; Lim, Choi, Lee, Han, et al., 2019), nanoplasmon‐enhanced scattering and dark field microscopy (Wan et al., 2019), as well as surface‐enhanced Raman scattering (Kwizera et al., 2018), among others. These may prove powerful techniques to expand EV research and its clinical applications, including point‐of‐care testing for diagnostics.

### 2.5 EV isolation based on microfluidic technologies

Microfluidic systems can be defined as integrated systems possessing two or more devices assembled into parallel autonomous operation. Usually, one or more devices are units composed of a network of microchannels, which can be interconnected, and are able to handle small volumes of media (Gholizadeh et al., 2017; Narayanamurthy et al., 2020). Because of this ability, microfluidic devices can often reproduce complex analytical processes on a microscale, with high accuracy and specificity. Additional specialized elements can then be added for facilitating fluid movement or expanding the number of available analyses (Guo et al., 2018).

Microfluidic‐based technologies are used for a myriad of applications, and have more recently been applied to EV research. These methods tap into both the physical and biochemical properties of EVs at microscales and usually combine high throughput with molecular detection capacity (Z. Chen, Yang, et al., 2020; Iliescu et al., 2019). Just recently, microfluidic analysis using as little as 2 μl of plasma (with no further processing) successfully detected tumor‐associated biomarkers in a subpopulation of EVs (P. Zhang et al., 2019), attesting to the potentially ultrasensitive power of microfluidics‐based technologies.

At present, EV isolation methods that are based on microfluidics are classified into three categories: size‐based, immunoaffinity‐based, or dynamic. Moreover, size‐based EV separation devices include nanofilters, nanoarrays, and nanoporous membranes (Iliescu et al., 2019). The latter has been shown to constitute a simple strategy to isolate vesicles from whole blood samples. A study by Davies et al. (2012) showed that EVs can be isolated directly from whole blood filtered through a nanoporous membrane into a microfluidic device using electrophoresis to force the vesicles through the membrane, thus increasing the efficiency of the isolation. A similar design by Rho et al. (2013), using negative pressure instead of an electrical current to drive the blood in the microfluidic circuit, proved to be less specific and less sensitive.

Nanofilter and nanoarray systems have been used in conjunction with unique mechanisms developed around microfluidic systems, aiming to address the issues of sample volume, reagent consumption, overall cost, and processing time that often surround EV isolation protocols. These mechanisms include acoustic manipulation (K. Lee et al., 2015; M. Wu et al., 2017); nanowire‐based traps (Lim, Choi, Lee, Kim, et al., 2019; Z. Wang et al., 2013); nano‐sized deterministic lateral displacement (J. T. Smith et al., 2018); viscoelastic flow (Liu et al., 2017); and microscale nuclear magnetic resonance (Shao et al., 2012).

Despite the potential of microfluidics‐based technologies to isolate and analyze EVs and EV components in an integrated and user‐friendly manner (Bernstein et al., 2021), there are still some problems to be resolved, namely those concerning the viscosity of some biological samples (which can block the microfluidic channels) and the small sample volumes used (which can be detrimental in cases where the target protein or biomarker is expressed in low concentrations).

### 2.6 Commercial kits for EV isolation

Several companies have developed quick and easy EV isolation kits in order to minimize the limitations of time and sample volume of the conventional methods; however, the reliability and specificity of these kits vary and they are not always the most economical solution. Generally, commercial kits for EV isolation/analysis tend to be relatively expensive, with the added constraint of only allowing analysis of a small number of samples.

Enderle et al. (2015) compared the RNA yield of EVs isolated using conventional ultracentrifugation and the commercial ExoRNeasy Serum/Plasma Maxi Kit (QIAGEN, Germany), which is an immunoaffinity kit. Results showed no difference in the concentration of intact vesicles recovered by the two methods, and the RNA yield obtained was also equivalent. Balaj et al. (2015) compared the recovery rate and RNA yield of EVs isolated by a protocol developed in‐house (using heparinized agarose) against conventional ultracentrifugation, and also against the ExoQuick™‐ТС commercial kit (System Bioscience) which is a polymeric precipitation kit. Results showed that the vesicles isolated using heparinized agarose were morphologically similar to those obtained by standard ultracentrifugation and the RNA content did not significantly differ between the three methods. Paolini et al. (2016) tested the purity and biological function of EVs isolated by simple and gradient ultracentrifugation against those isolated by the commercial one‐step precipitation kit Exo PK (Invitrogen). Their results showed that the preparations obtained by gradient centrifugation were of higher purity and biological activity.

In summary, while commercially available kits represent an attractive alternative for the quick and less labor‐intensive isolation of EVs, problems concerning their present lack of specificity will need to be addressed before they can be routinely used.

### 2.7 Other methods and future perspectives for EV isolation

Due to the explosion of interest in the study and application of EVs as novel therapeutic targets, biomarkers and nano‐sized biocompatible drug carriers, among other uses (Bazzan et al., 2021), innovative advances in the development of techniques for the isolation of these vesicles are frequently being reported in the literature. For example, a strategy for the specific isolation of EVs from relatively small volumes of human serum has recently been proposed which takes advantage of the naturally reversible, high affinity binding between titanium oxide and the phosphate groups on the surface of the lipid bilayer of EVs (Gao et al., 2019). This protocol produced a high‐purity isolation with good recovery of EVs which could then either be eluted to obtain intact EVs or directly lysed for downstream proteomic analysis.

## 3 CURRENT METHODS FOR THE CHARACTERIZATION OF EVs

After isolation, EVs are often characterized by size, concentration, presence of protein markers, protein concentration, and other components. However, not all types of characterization are necessarily included in all studies and data obtained by different methods can differ significantly. These factors, associated with the lack of sufficient methodological information, make it difficult to compare different studies within the field (Théry et al., 2018).

As with other groups, we believe that existing EV isolation techniques, the majority of which are used in basic research, will become more efficient when they begin to be used in clinical applications (Gowen et al., 2020; Inal, 2020; O. Wiklander et al., 2019), particularly if isolation techniques are coupled with integrated multiplexed analysis able to perform EV analyses. This still remains a major challenge as, despite the great advances in EV isolation methodology, techniques that allow efficient, quick and cost‐effective detection, quantification, and characterization/analysis of EVs are lagging behind. Accordingly, EV characterization is still a matter of debate and remains a challenge, no consensus, for example, yet being reached about EV‐specific markers.

**FIGURE 1:**
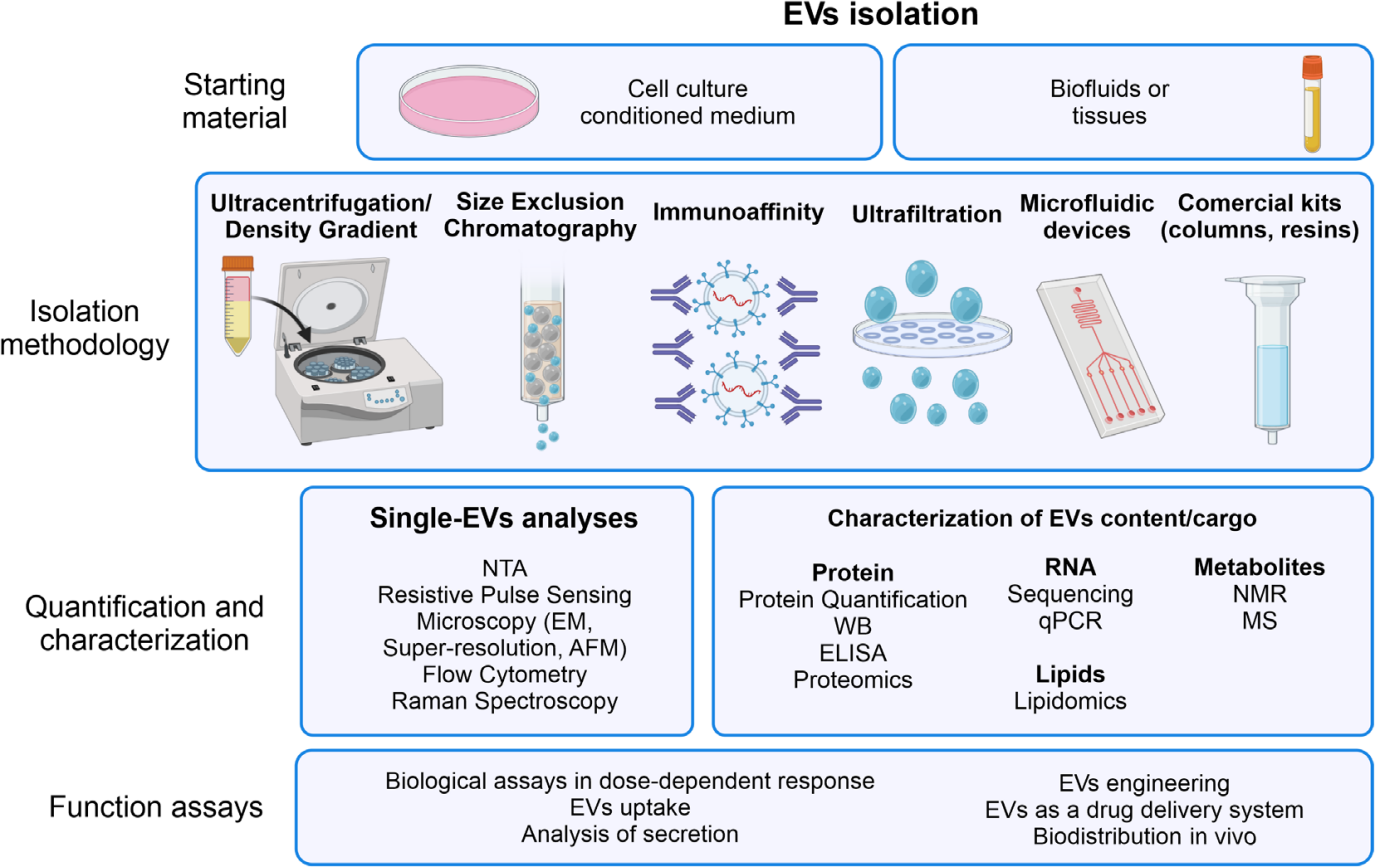
Schematic representation of the workflow for isolation and characterization of EVs, with the most used methods in each step. AFM, atomic force microscopy; EM, electron microscopy; MS, mass spectrometry; NMR, nuclear magnetic resonance; NTA, nanoparticle tracking analysis; WB, Western blotting. Image created using BioRender (https://app.biorender.com/)

The methodologies used to characterize EVs by quantity/abundance, size, or content/composition vary according to the subsequent analyses. Figure 1 outlines a flow of the study of EVs, from isolation to their characterization by various approaches. Interestingly, much work has been dedicated to comparing different methodologies, reinforcing the importance of technical knowledge of the instruments, awareness of analytical variables, and recognition of instrument settings when analyzing EV populations (Akers et al., 2016; Erdbrügger & Lannigan, 2016). In this section, we will consider the most used techniques and their potential application in characterizing EVs (Gardiner et al., 2016) in terms of physical and biochemical characteristics. As the methods are not specific for EV subtypes (MVs or exosomes), the term “EVs” will be used to cover all populations. In addition, these methods are not exclusive to the analysis of EVs, but adapted for this field, meaning there is a large scope for optimization to enable adequate evaluation of EVs.

### 3.1 Single EV analysis

The characterization of EVs with their small size and low refractive index makes detection by light scattering methods, such as conventional flow cytometry difficult. In addition, they are heterogeneous in size, and there may be interference from lipoproteins and protein aggregates of similar size. The antigen density in EVs is often low and a very large amount of EVs may be required to be detectable by techniques such as ELISA and western blotting. Thus, single‐particle analysis can help further delineate heterogeneous and complex populations of EVs into subgroups better defined by physicochemical and molecular characteristics. The main single‐particle analysis techniques in the field of EVs are nanoparticle tracking analysis (NTA), microscopy (electron microscopy [EM], cryo‐EM, atomic force microscopy, and high‐resolution microscopy), resistive pulse sensing, high‐resolution flow cytometry, and Raman spectroscopy (Chiang & Chen, 2019).

#### 3.1.1 Nanoparticle tracking analysis

NTA is one of the most used methods in EV research, as it provides parameters of concentration and particle size. This technique combines laser light scattering microscopy with a camera, which allows the visualization and recording of nanoparticles in solution. In this system, vesicles of about 30–1 μm are driven by a flow and the NTA software tracks the Brownian motion of individual particles and calculates their size and total concentration. This technique has been particularly important to assess the release of subpopulations of EVs, generally categorized into lEVs and sEVs, respectively (Crescitelli et al., 2020; Durcin et al., 2017; Gavinho et al., 2020; Théry et al., 2018; Yekula et al., 2020).

The NanoSight instrument incorporates a laser and camera for detecting fluorescent particles. This property has been exploited to determine the phenotype of vesicle subpopulations according to their labeling with specific antibodies or fluorescent markers (Desgeorges et al., 2020; Dragovic et al., 2011; Thane et al., 2019). Furthermore, Baldwin et al. (2017) showed that it is possible to use this fluorescence property of NTA to determine the content of miR‐21 targets contained in lung cancer‐derived EVs and their stoichiometry in relation to the total EV population. The assay was designed using a mixture of tumor cell‐derived EVs that fuse with cationic lipoplex nanoparticles, the latter containing (encapsulated) fluorescently labeled microRNAs (miRNA)‐specific molecular beacons. These are fluorophore quenched oligonucleotide hybridization probes whose fluorescence is restored upon binding to a target nucleic acid sequence. After mixing the EVs with the particles carrying the molecular beacons, a combination of light scattering and fluorescence nanoparticle tracking was used to identify the proportion of the total EV population that contained the target miRNA transcripts. This type of experiment opens new avenues for the identification of specific targets carried by vesicles and presents a more sensitive alternative than cytometry, which loses sensitivity with vesicles below 100 nm, such as most exosomes (Pasalic et al., 2016).

#### 3.1.2 Microscopy techniques for EV imaging

EV imaging helps researchers to understand physical properties of EVs, their morphology, mechanism of release and uptake, and enables detection of biomarkers expressed on the EV surface (Fertig et al., 2014; Han et al., 2021; Höög & Lötvall, 2015; Morelli et al., 2004; Sorrells et al., 2021; Szempruch et al., 2016). EM, for example, played a key role in the first descriptions of the presence of MVBs inside cells (Harding et al., 1983) and has still been used in the field of EV investigation due to the images of high resolution (ranging from 1 to 3 nm for transmission electron microscopy (TEM) and approximately 5 nm for scanning EM). In addition, using immunogold‐labeling, TEM can further reveal EV proteins, which can help in the understanding of the role of these vesicles (Marcilla et al., 2012; Shi et al., 2019).

The electron microscope has, however, its drawbacks, which include loss of material during extensive sample preparation, lack of multiparametric phenotyping and low throughput capacity. In the field of EVs, one of the problems of this methodology is the possible alteration in morphology, particularly of exosomes during preparation, resulting in the characteristic “cup‐shape” (Lobb et al., 2015). This has been suggested to be an artifact caused by sample dehydration, since cryo‐EM results have shown that EVs have a perfectly spherical structure in aqueous solution (Conde‐Vancells et al., 2008; Kadiu et al., 2012). Indeed, the preparative steps of chemical fixation, dehydration, observation under vacuum, and electron beam radiation damage could all interfere with an important feature of EVs. Some alternatives have been proposed, including the use of protein‐rich material (such as matrigel or albumin) or some inert polysaccharides (such as agarose or methylcellulose) to protect against material loss and keep the EVs preserved during the fixation processes. In addition, some groups have used negatively stained whole mount preparation, in which EVs are adsorbed onto a metal grid, chemically fixed and negatively stained prior to observation (Ramirez et al., 2018).

As a result, there has been a great advance in microscopy techniques that are being explored for the visualization of EVs, such as cryo‐EM, electron tomography EM, atomic force microscopy (AFM), confocal microscopy, and super resolution microscopy, each with their own resolving power and specific advantages and disadvantages (reviewed by Chuo et al., 2018).

Cryo‐EM does not use staining or chemical fixation procedures and samples are directly applied onto an EM grid, vitrified and visualized. In this preparation, water is transformed into a glass‐like state without the formation of ice crystals. Cryo‐immobilizing allows biological structures to be preserved in their native hydrated state, thus avoiding artifacts commonly caused by conventional fixation. Cryo‐EM also allows 3D tomographic data collection thus enabling the spatial visualization of more complex structures (Höög & Lötvall, 2015; Votteler et al., 2016; Yang et al., 2020; Yuana et al., 2013). Similar to conventional TEM, cryo‐EM can be combined with immunogold labeling, providing data on the presence of certain molecules carried by EVs (Brisson et al., 2017; Goughnour et al., 2020).

AFM uses a microscopic physical probe to scan through the surface of specimens and detects the morphology of the sample in three‐dimensional space, obtaining information from the very weak interaction between the probe and the sample surface. By recording the probe position during the scan, AFM generates topographic images of the samples with a resolution limit around 1 nm, which allows imaging of most EVs (Bairamukov et al., 2021; Kim et al., 2019).

The term super resolution microscopy refers to an assortment of various imaging approaches including single‐molecule localization microscopy, which encompasses both stochastic optical reconstruction microscopy and photo‐activated localization microscopy. In conventional fluorescence microscopy, every fluorophore in a sample is activated, meaning that closely grouped fluorescence emitters cannot be resolved due to overlapping point‐spread functions. Single‐molecule localization microscopy exploits the temporal gap between activation‐deactivation cycles of nearby molecules to detect their spatial separation (F. Colombo et al., 2021). As a result, it is possible to reconstruct the position of each emitter with a spatial resolution of ≤20 nm by combining thousands of frames (Rust et al., 2006). It was shown that different fluorescent dyes or conjugated antibodies can provide a good definition of EVs using this technique (C. Chen et al., 2016; Nizamudeen et al., 2018; Skovronova et al., 2021). This methodology shows a great sensitivity for EVs, where cancer‐specific markers can be detected on single EVs isolated from body fluids, a very difficult detection by conventional techniques.

#### 3.1.3 EV concentration, size, and charge by resistive pulse sensing

Tunable resistive pulse sensing (TRPS) provides reliable and fast particle‐by‐particle measurement of EV size and concentration distribution. In TRPS, a tunable submicron‐sized pore separates two fluid chambers, one containing the sample to be analyzed, the other an electrolyte solution. By applying a voltage across the membrane, a flow of ions is induced. Once a particle moves through the nanopore, the flow of ions is altered resulting in a brief “resistive pulse” which is recorded by the instrument. When a particle passes through the pore causing a change in voltage, the magnitude of this pulse is proportional to the volume of the particle traversing the pore, and the blockade rate is directly related to the particle concentration (Maas et al., 2017; Vogel et al., 2016).

An adaptation of this technique is microfluidic resistive pulse sensing (MRPS). MRPS differentiates itself from TRPS in that MRPS uses cartridges that have rigid, precalibrated pores made of polydimethylsiloxane, while TRPS uses sensing pores of polyurethane that can be stretched to tune the pore size. MRPS allows control flow of samples through the constriction using controlled pressure (Cimorelli et al., 2021). Some protocols are already available for measuring EVs using variations of the resistive pulse sensing technique (Coumans et al., 2014; Maas et al., 2014; Vogel et al., 2016) with equipment available on the market, for example, IZON qNano (Izon Science Ltd) and nCS1™ instrument (Spectradyne LLC).

#### 3.1.4 Single EV analysis by flow cytometry

One method for high‐throughput multi‐parametric analysis and quantification of EVs is flow cytometry. Flow cytometry is a technology that records both the scattering and fluorescence signals generated by individual particles as they are illuminated by a laser beam while passing through a nozzle. The intensity of detected light is reported as forward light scatter and side light scatter (SLS). The quantity of light scattered forward is proportional to the diameter, while SLS indicates morphology and inner anatomy of EVs (Lannigan & Erdbruegger, 2017; Lucchetti et al., 2020). One of the great advantages of flow cytometry is the ability to analyze multiple labels on individual particles and to identify various types and subsets. Despite its wide application, some limiting factors make it difficult to use in the field of EVs. These include the small size of EVs, resulting in a low sensitivity to discriminate them using most popular flow cytometry equipment (or even to discriminate them from background signals). This low fluorescence being emitted by labeled EVs is due to the low number of antigens per particle and limited feasibility of post‐stain washing to reduce background fluorescence (Inglis et al., 2015; Lannigan & Erdbruegger, 2017). Therefore, the correct application of cytometry often requires special equipment or at least specific components for the detection of small particles, such as beads. Despite this, some studies have brought solutions to these problems and optimized systems and protocols can elevate the detection limit, as commented later in this article under future directions (D. Choi et al., 2019; Görgens et al., 2019; Morales‐Kastresana et al., 2017; Nolan & Duggan, 2018; Y. Tian et al., 2018).

#### 3.1.5 EV purity and composition by Raman spectroscopy

Vibrational spectroscopies, and in particular Raman spectroscopy are attractive tools for the study of EVs providing an overall biochemical characterization without labeling or targeting any previously known marker at the single‐particle level (K. Lee, Fraser, et al., 2018; Morasso et al., 2020). A source of high‐intensity light is applied to the sample, and incident photons are scattered by molecules. The frequency and intensity of scattered radiations reveal the quality and quantity of the sample, respectively (Butler et al., 2016). An interesting study showed that, by means of Raman spectroscopy, a fingerprint of the amyloid‐beta (Aβ) peptide (one of the hallmarks of Alzheimer's disease) was present in the cargo of sEVs derived from a cell culture model and from midbrain organoids. These findings open a positive path for the investigation of EVs in the identification of biomarkers of neurological disorders, such as toxic proteins (Imanbekova et al., 2021).

One approach that has been used in the field of nanoparticles is laser Tweezer Raman spectroscopy, which combines optical trapping with Raman probing. Optical tweezers consist of using a diffraction limited beam to stably trap a particle in three dimensions creating a net force that brings them to the axial center of the incident laser (Enciso‐Martinez et al., 2020). This technique has already been used to characterize EVs in several biological models (Kruglik et al., 2019; Z. J. Smith et al., 2015; Tatischeff et al., 2012).

### 3.2 Characterization of EV content

#### 3.2.1 Protein content of EVs

Proteins present in EVs may provide clues about biological functions and their effects in cell communication. Therefore, many groups have chosen to characterize EVs from the point of view of proteins. Initially, a basic method can be applied to quantify the total protein content, such as the Coomassie Brilliant Blue G‐250 assay (also called Bradford assay) or the bicinchoninic acid assay (Théry et al., 2018). Both rely on quantification of proteins by absorbance measurement of a colored complex between a reagent and the protein, based on a calibration curve of known concentrations. EV total protein dosage data can be associated with other applied characterization methods, providing interesting correlations such as the protein per nanoparticle ratio (De Sousa et al., 2022). It is important to emphasize that problems in the purification of EVs will impact the amount of proteins dosed, since contaminants can be measured and generate a bias. In addition, it must be ensured that the EVs are broken (by the action of detergents or freeze–thaw cycles) prior to dosing in order to have a total value of proteins both inside and in the membrane of the vesicles.

The search for EV markers is extensive, but fails to differentiate subpopulations of particles. Some of the markers that had been suggested in past years (such as HSP70, flotillin‐1, TSG101, CD63) are not present in every/each vesicle or can be found in both sEV and lEV (M. Colombo et al., 2013; Crescitelli et al., 2020; Kowal et al., 2016; Yoshioka et al., 2013). Due to this, there is no single protein or combination of proteins that can be recommended as universal EVs markers. The demonstration of the enrichment of molecules present in EVs and absence (or depletion) of putative contaminants is a good alternative in the biochemical characterization of EVs. The lack of specific markers brings a change in the naming of EVs: unless authors can establish specific markers of subcellular origin that are reliable in their experimental systems, authors should consider using “operational terms” for EV subtypes, as referred to earlier in this review (Théry et al., 2018).

Proteomics technologies have provided a significant contribution to the field of EVs, allowing the creation of large‐scale profiling of proteins secreted through EVs, which can be confirmed later with other methodologies, such as western blotting. Several works have used proteomics to identify differences in the EVs of biological samples from patients and to quantify the presence of some peptides compared to healthy individuals. Notably, one study showed that EVs derived from the serum of breast cancer patients can differentiate the molecular subtypes of breast cancer (such as triple‐negative or *HER2*) using a proteomic approach (Rontogianni et al., 2019). This shows the prospect of their use as non‐invasive biopsies for diagnosis and management of cancer patients.

#### 3.2.2 RNA content of EVs

The presence of RNA inside EVs has attracted the attention of researchers since these nucleic acids are protected from degradation in the extracellular environment and can be delivered intact to recipient cells (Hinger et al., 2018; O'Brien et al., 2020), even between different species (or kingdoms) (Stanton, 2021). These RNA populations include various protein‐coding transcripts (mRNAs) and many types of noncoding RNAs, including miRNAs, long noncoding RNAs, circular RNAs, small nucleolar RNAs, small nuclear RNAs, transfer RNAs, ribosomal RNAs, and piwi‐interacting RNAs (O'Brien et al., 2020). Many groups have been dedicated to understanding the mechanisms of this RNA packing/loading within EVs and the differences in RNA profiles between different cell types or conditions (Ge et al., 2019; Y. Li et al., 2019). The RNA content varies greatly according to the physiological state of the cells and differs substantially from the cellular RNA content (in types of RNA and relative concentrations of specific RNA sequences) (Baglio et al., 2015; Bayer‐Santos et al., 2014; Guduric‐Fuchs et al., 2012; Skog et al., 2008). Some database and community‐contributed catalogues of molecules identified in EVs have emerged in the past years, such as Vesiclepedia (Kalra et al., 2012), Exocarta (Mathivanan & Simpson, 2009), exRNA (Murillo et al., 2019), and ExoRBase (Lai et al., 2022).

Some difficulties arise in establishing the functionality of RNA in EVs. Firstly, overexpression systems that increase the amounts of a particular RNA in EVs can result in supraphysiological levels of the RNA in the source cells, affecting their physiological behavior (O'Brien et al., 2020). Also, contaminating EVs, such as those present in fetal bovine serum, can also carry RNA and confuse interpretations. To avoid the contamination with non‐intravesicular RNAs, ISEV recommends performing a proteinase and RNAse treatment before RNA extraction. In the same way as for other characterization approaches, the methodology of isolation of EVs can influence the purity of the material found and the RNA content.

The RNA content of EVs has been studied extensively using high‐throughput RNA‐Seq and is commonly validated using reverse transcriptase quantitative polymerase chain reaction (RT‐qPCR) analysis (Everaert et al., 2019). One of the difficulties of working with EV‐derived RNA is the low yield of material, which is often below the detection limit of the most common quantification techniques, such as fluorimetry. This can be overcome by vacuum concentrating all the RNA extracted from EVs prior to analysis.

There is a concerted effort to find diagnostic and prognostic markers based on the detection of mRNAs in EVs. Detection of these biomarkers in biofluids in different disorders will avoid more invasive tests (Castellanos‐Rizaldos et al., 2018; Cha et al., 2020; de Gonzalo‐Calvo et al., 2016). In fact, these advances are already present in the clinic today, for example, for the prognostic evaluation of prostate tumors with urine samples (Bio‐Test Prostate Techne ExoDx; Tutrone et al., 2020).

#### 3.2.3 Lipid content of EVs

Lipids are relevant components of EVs, constituting a significant fraction of the total EV volume (especially in sEVs), considering a membrane thickness of about 5 nm (Kreimer et al., 2015). Lipids in EVs form a protective barrier for their cargo and can carry markers derived from their cell of origin. They also participate in membrane fusion events and biomolecule delivery. Hundreds of lipid varieties have been identified in EVs in several reports; cholesterol, phosphatidylcholine, phosphatidylserine, and sphingomyelin derivatives are among the most commonly detected (Brzozowski et al., 2018; Haraszti et al., 2016; Llorente et al., 2013; Skotland et al., 2020). EVs tend to have a higher lipid:protein ratio and a different lipid content than their parent cells (Haraszti et al., 2016; Llorente et al., 2013; Lydic et al., 2015; Skotland et al., 2019; Sun et al., 2019; Subra et al., 2007). The description of both the relative and the absolute values of lipid classes in different EVs provides interesting information about the composition of these particles and can be explored through quantitative lipidomics (S. Chen et al., 2019; Sun et al., 2019). There are several reports describing how to perform quantitative lipidomic studies and the recommended extraction methods. Also, there are some methods used for total quantification of lipids in EVs (such as sulfo‐phospho‐vanillin assay [SPVA], fluorescent dyes that incorporate into membrane bilayers or Fourier transform infrared spectroscopy), but they have their limitations in sensitivity for detection of some types of lipids (Théry et al., 2018).

Brzozowski et al. (2018) identified differences in the relative abundance of lipid species in EVs derived from three prostate cell lines (one non‐tumorigenic, one tumorigenic and another metastatic) by the approach of quantitation of molecular lipid species by target lipidomics. Other work also found differences in the lipid composition of EVs derived from different breast cancer cell lines and showed that the contents differed from their cell of origin (Nishida‐Aoki, Izumi, et al., 2020).

#### 3.2.4 Metabolite content of EVs

Metabolites are a type of small molecule (with a molecular weight <2 kDa) being the downstream products of various biological reactions. They can be steroid hormones, amino acids, metabolic intermediates of nutrient and lipid anabolism, among other molecular species. Despite participating in almost all cellular processes, these are the least studied components of EVs to date.

Two main analytical methodologies are mostly applied for characterizing metabolites in EVs: nuclear magnetic resonance (NMR) spectroscopy and high‐resolution MS. Čuperlović‐Culf et al. (2020) evaluated the metabolome of sEVs derived from different glioblastoma cells by NMR spectroscopy. A clear difference was seen between the metabolic profiles of the cells when comparing EVs and conditioned media. Another study performed a metabolomic analysis of EVs derived from pancreatic cancer cells (PANC‐1) cultured under different oxygen concentrations, as hypoxia contributes to the malignant behavior of these cells (Hayasaka et al., 2021). This work also showed that the metabolite profile of the EVs were different in relation to the cell of origin. A total of 140 hydrophilic metabolites were detected in sEVs and it was observed that the metabolomic profile of sEVs changed under hypoxic stress and that there was an increase in the metabolites involved in angiogenesis.

## 4 FUNCTIONAL ASSAYS AND BIOLOGICAL CHARACTERIZATION OF EVs TOWARD DEVELOPING EVs AS THERAPEUTICS

Although the physical and biochemical characterization of EVs is important, only functional assays can provide answers about the real role of EVs in biological systems. The study of EVs can take place both in vitro and in vivo and has been explored in different areas of knowledge from basic cell biology to immunology, pathology, among others. Certain questions can guide the definition of an appropriate model to test hypotheses about EVs: (i) What is the origin of EVs and what is the secretion stimulus? (ii) What are the approximate physiological amounts of secreted EVs? (iii) Which EV populations are being evaluated (e.g., only lEVs, only sEVs or only EVs containing a certain epitope)? (iv) Which tests can provide answers about the role of EVs in this biological model?

**FIGURE 2:**
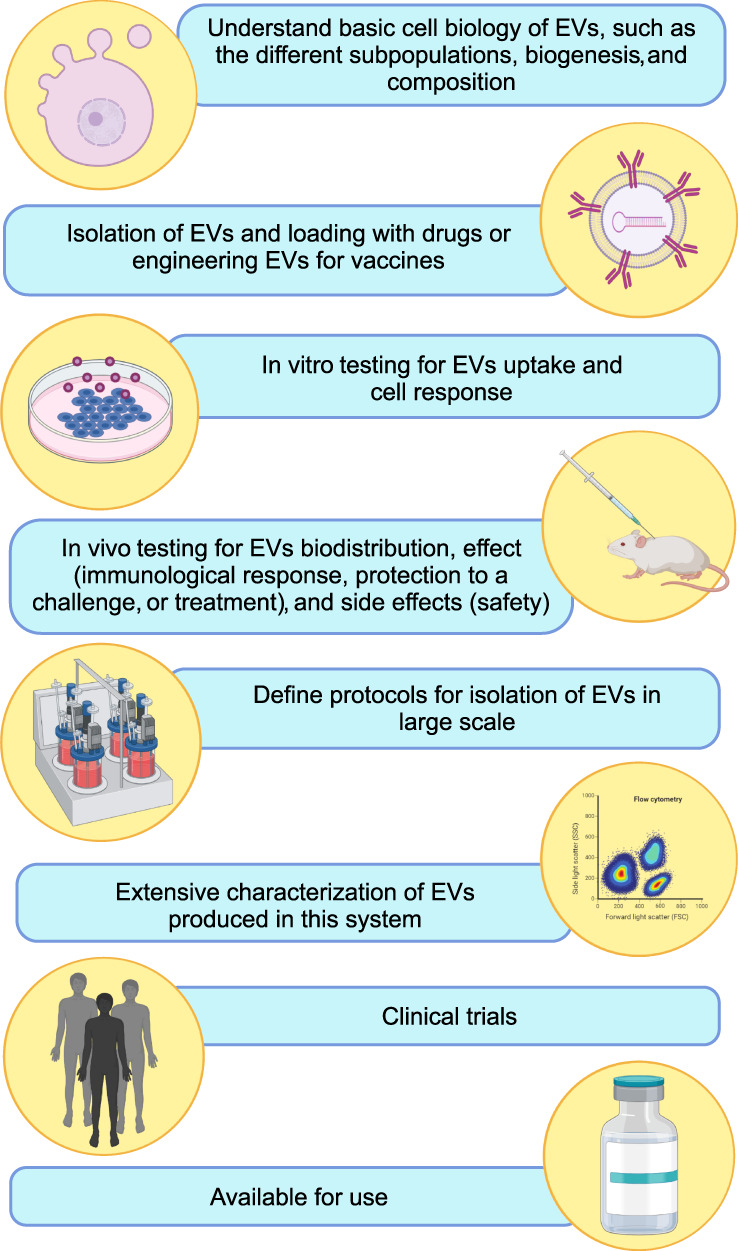
Steps toward the translational application of extracellular vesicles (EVs), from basic research to market arrival. Image created using BioRender (https://app.biorender.com/)

In most cases, the EVs are studied within limited conditions and the results cannot always be extrapolated to other models. Also, it is important to emphasize the need for adequate controls for each experiment using, for example, EVs from healthy cells or cells not exposed to treatments. Figure 2 shows the long path from the characterization of EVs toward their translational application.

### 4.1 Assays to ascertain functional capacity of EVs, toward developing EV‐based therapies

#### 4.1.1 Biological responses modulated by EVs

In the EV field, there are a range of biological assays which have been used to understand the function of EVs and their suitability for therapeutic use (Nguyen et al., 2020). These include assays for inducing transformation and differentiation in cells (Ansa‐Addo et al., 2010; Ismail et al., 2013), change in permissiveness to infection by pathogens, cell migration, and changes in cytokine release or triggering of secondary signals. It is further recommended that these trials be performed on progressive doses of EVs to show dose dependence.

#### 4.1.2 Cellular uptake of EVs

Cells appear to take up EVs by a variety of pathways, including clathrin‐dependent endocytosis, caveolin‐mediated endocytosis, macropinocytosis, phagocytosis, and direct fusion to PM (Mulcahy et al., 2014). Indeed, it seems likely that a heterogeneous population of EVs may gain entry into a cell via more than one route. The analysis of the capture of fluorescently labeled EVs in the presence of a series of drugs that inhibit these internalization pathways can clarify the EV uptake mechanism and also monitor their intracellular trafficking after entry (Lange et al., 2016).

#### 4.1.3 In vivo biodistribution of EVs

Understanding the in vivo fate of EVs is of utmost importance for future therapeutic applications. Using different approaches, it was shown that the biodistribution of EVs was influenced by the route of administration and the origin of the EVs (Gupta et al., 2020; Nishida‐Aoki, Tominaga, et al., 2020; Royo et al., 2019; O. P. Wiklander et al., 2015). Using EVs fluorescently labeled with DiR (1,1'‐Dioctadecyl‐3,3,3',3'‐Tetramethylindotricarbocyanine Iodide) or CD63‐EGFP‐labeled EVs, O. P. Wiklander et al. (2015) showed that 24 h after EV injection, the main sites with detected fluorescence were the liver, spleen, lungs, and gastrointestinal tract. This study also evaluated the targeting of EVs derived from cells transfected with the fusion protein LAMP2b‐RVG which targets the Rabies virus glycoprotein (RVG) peptide to the EV membrane (RVG‐coupled species are more readily addressed to the brain, due to the presence of nicotinic acetylcholine receptor). The DiR‐labeled EVs were injected intravenously into the animals and, interestingly, the RVG‐EVs displayed a significantly increased signal in the brain compared to the non‐RVG (mock) EV group. This work has also shown that there is a propensity for tumor tissues to capture injected EVs, possibly due to the high vascularization of the tumor and the permeability of the vasculature in the region(Wiklander et al., 2015). This opens a possibility for anti‐tumor therapies delivered by EVs, especially if optimized using different cell sources and target inclusion.

Royo et al. (2019) showed that EVs derived from proliferative liver cells accumulate mainly in the liver and lungs in mice after a short period (15 min). After treating the EVs with neuraminidase (an enzyme that digests the terminal sialic acid residues of glycoproteins), their accumulation in the lungs was greater than that of untreated EVs. Furthermore, when the EVs were injected through the hock, the neuraminidase‐treated vesicles distributed better to the axillary lymph nodes than the untreated EVs.

#### 4.1.4 EVs as a drug delivery system

EVs have features that qualify them as a potential drug delivery system, some of which have been previously described. These include that EVs carry and protect a wide range of biomolecules and are able to deliver them to recipient cells; they are distributed through the circulation with a certain stability to reach different organs, including crossing the blood–brain barrier. They can be engineered to optimize delivery to certain tissues and have a high level of biocompatibility (Elsharkasy et al., 2020). Despite its advantages, there are technical obstacles to the use of EVs in the clinic, such as the ability to upscale the production and isolation of EVs. Escudé Martinez de Castilla et al. (2021) provide a very complete systematic review of preclinical studies involving EVs as drug carriers, including the most used procedures for encapsulation of active pharmaceutical ingredients in EVs, routes of administration and purity estimation. Greater understanding of EVs, as well as comparative analysis of EVs to other synthetic delivery vehicles (such as liposomes, lipid nanoparticles, and polymeric micelles) can provide answers about the future use of EVs as a drug delivery system (Moore et al., 2017).

## 5 FUTURE DIRECTIONS FOR EV ISOLATION AND CHARACTERIZATION

It is expected that further development of the methods currently in use for EV isolation and characterization will allow for ever more detailed dissection of EV biogenesis, and promote a better understanding of their content and functional properties. Each technique presents benefits and weaknesses, and the selection of any particular isolation method presents a multifactorial problem. It is therefore worth considering the optimization of certain methods to, for example, improve detection limits and resolution.

### 5.1 Optimization of flow cytometric techniques for EV analysis

Recent research has led to the construction of customized high‐sensitivity flow cytometers that can detect single vesicles, as well as enumerate, size, and recognize molecular markers. A high‐sensitivity flow cytometer was reported by Stoner et al. (2016) using parts from commercially available equipment and integrated optimized sample preparation using fluorescent membrane staining. This method termed vesicle flow cytometry (VFC) combined an optical bench, photomultiplier tube detector and fluorescence detector (Stoner et al., 2016). This custom‐made VFC was used to assess several fluorescent probes and determined that the voltage sensing dye di‐8‐ANEPPS emitted fluorescence in relation to the surface area of the vesicle enabling the measurement of its size and concentration. The estimated size of vesicles identified using this method was in the range of 70–80 nm, and the authors theorized that an increase in sensitivity could reduce the detection limit to <40 nm. Nevertheless, it was found that the VFC results regarding vesicle size and concentration were in agreement with those obtained by NTA. VFC could also detect a subpopulation of CD61‐positive vesicles from platelet‐rich plasma demonstrating a greater resolution attributed to increased laser power, decreased flow, and increased duration of signal integration (Stoner et al., 2016).

Further optimization of flow cytometric instruments has been reported for the detection and quantification of single particles using fluorescence‐labeled vesicles for calibration, rather than submicron sizing beads. Imaging flow cytometry (IFCM) integrates imaging cameras with conventional flow cytometers (Headland et al., 2014). The signals generated with IFCM are examined through a digital microscope to produce quantitative imaging data detected with a charged coupled device camera. Görgens et al. (2019) reported an optimized IFCM method. Using enhanced green fluorescent protein (eGFP) fused with CD63‐labeled vesicles derived from monocytic THP‐1 cells, eGFP‐CD63 vesicles were used as a reference material to modify various parameters to increase the contrast between background noise and vesicle signals. At present, researchers use polystyrene and silica beads of known sizes to calibrate flow cytometers for the detection of EVs but while this has provided superior and consistent flow cytometric methods in EVs analysis, the beads lack the physical characteristics that EVs possess such as different refractive indices. This optimized IFCM method was shown to detect and quantify single vesicles and vesicle subpopulations from a heterogeneous population without the need of prior vesicle purification or isolation of samples.

Nano‐flow cytometry (NFCM) is an emerging technology that can detect nanosized particles such as viruses and EVs. It can detect, in real time, light elastically scattered by single nanosized silica and gold particles as small as 24 and 7 nm in diameter, respectively (S. Zhu et al., 2014). The high sensitivity of NFCM was attributed to a decrease in the sample volume, the reduced sample stream diameter and reduced size of the laser beam contact site, as well as the higher laser energy and an increased particle contact time with the laser beam. As a recent application of NFCM it has been used to define subpopulations of EVs from cancer cells (D. Choi et al., 2019) and in nanomedicine to determine the size and content of individual liposomes carrying doxorubicin (C. Chen et al., 2021). Simultaneous fluorescence detection allowed for the measurement of the size distribution and payload of individual liposomes and the results showed variations in vesicle size and content.

Ma et al. (2016) used NFCM for a label‐free detection of viruses, with diameters of 27 nm, as well as distinguishing them in a mixture, through the quantification of ultraweak elastically scattered light from single‐virus particles. NFCM was also able to identify distinct physical differences of the viral particle such as the position of tail fibers of the bacteriophage PP01, distinguishing empty capsids from mature virions and the content of capsids with and without enclosed DNA. This technology is particularly important for distinguishing virions and similarly sized EVs.

### 5.2 Emerging techniques for EV analysis

The potential application of EVs in medicine (particularly nanomedicine) is clear but their use in a clinical setting is still hampered by the limitations of methods currently available, for vesicle isolation, detection, and characterization. The new technologies being developed seek to integrate different approaches, especially those based in antibody labeling or capturing. Advances in the analytical tools used in EV research are leading to improvements in generated data, particularly data that can characterize individual vesicle details such as size, concentration, and protein expression. Efforts are also directed to create inexpensive, rapid, reproducible assays that can be applied to clinical settings without the need for extensive preparation of EVs from bodily fluids. One promising field is biosensing technology that seeks to combine current conventional methods with lab‐on‐chip devices to facilitate EV analysis. Biosensors are analytical instruments that detect and/or quantify biomarkers by producing signals related to the concentration of an analyte (Bhalla et al., 2018). These biosensors are composed of a biological receptor that provides specificity and a transducer that converts biological signals into electrical data (Naresh & Lee, 2021). Biosensors are generally grouped based on the type of transducer they use: electrochemical, fluorescence, or optical.

#### 5.2.1 Electrochemical biosensing systems for EV analysis

Electrochemical biosensing technology is an emerging field in EV analysis, with several variations employed. For example, an electrochemical sensor‐based system, named integrated magneto‐electrochemical sensor (iMEX) was reported to quickly isolate and detect exosomes in clinical samples (Jeong et al., 2016). The system is based on the specific isolation of exosomes with magnetic beads attached to anti‐CD63, CD81, and CD9 antibodies and the electrochemical detection of exosomal proteins (Jeong et al., 2016). It has eight detection channels each with a potentiostat to measure the electrical current. Individual potentiostats are attached to a digital‐to‐analog converter, analog‐to‐digital converter, a multiplexer, and a micro‐controller. The system was designed as a handheld unit with a value of <£50. It aims firstly to capture exosomes with magnetic beads followed by the binding of secondary antibody with horseradish peroxidase (HRP) and the addition of a chromogenic electron‐mediator, tetramethylbenzidine (TMB). This produces an electrical current which is then detected, enabling the iMEX system to detect exosomes at a sensitivity of <10^5^ from a minimal specimen volume of 10 μl and generating results in 1 h (Jeong et al., 2016). iMEX was customized to detect exosomes in blood from ovarian cancer patients. The selected markers, CD63, EpCAM, CD24, and CA125 along with their respective IgG‐controls were measured simultaneously. The results of the profiling of the plasma samples showed exosomes from cancer patients had higher levels of EpCAM and CD24 expression in comparison to non‐cancer patients. An advantage of the iMEX system is the detection of a subpopulation of exosomes from liquid samples, without the need for isolation methods such as ultracentrifugation or filtration (Jeong et al., 2016).

Electrochemical sensors can also be utilized in the characterization of the nucleic acid content of exosomes. Shao et al. (2015) described a microfluidic platform called the immuno‐magnetic exosome RNA (iMER) system which combines immunomagnetic exosome separation, RNA purification, and real‐time PCR. iMER operates based on the capture of exosomes with magnetic beads coated with anti‐EGFRvIII antibodies. After the addition of patient blood samples to the system only exosomes with the targeted markers are isolated on the chip. The captured exosomes are then lysed to release mRNAs that are then absorbed onto glass beads via electrostatic interactions. The mRNAs undergo amplification and quantitation by the on‐chip PCR. The iMER system was used to analyze the mRNA profiles of glioblastoma‐derived exosomes from human blood, glioblastoma cells, and used to assess the efficacy of drug treatment (Shao et al., 2015). The level of mRNA expression of O6‐methylguanine DNA methyltransferase and alkylpurine‐DNA‐N‐glycosylase enzymes involved in reversing DNA damage from temozolomide correlated with drug resistance (Shao et al., 2015). The iMER system has several advantages over other conventional methods including simplicity (quick turnaround time, typically 2 h), sensitivity, portability, and the ability to work with small specimen volumes (~100 μl).

#### 5.2.2 Surface plasmon resonance, quartz crystal microbalance, and opto‐fluidic smartphone for detection and characterization of EVs

Surface plasmon resonance (SPR) and quartz crystal microbalance (QCM) have common features including the use of gold film sensors to detect mass change that they are label‐free and that they transport sample in solvent across the sensor by means of a flow system. However, while SPR is an optical technique, mass changes being monitored based on a plasmonic principle (changes in light‐sensor interaction), the QCM measures change in mass based on the change in frequency of oscillations of the (gold‐plated) quartz crystal (thus, a mechanical technique).

SPR sensors represent a new method in EV detection and characterization with the advantage of label‐free detection and minimal sample processing. The SPR sensor‐based method called the nano‐plasmonic exosome (nPLEX) assay first developed by Im et al. (2014) showed the quantitative detection and characterization of exosomes from the ascitic fluid of ovarian cancer patients. nPLEX is centered on the transmission of SPR through periodic nanohole arrays located on an opaque gold film. The nanohole arrays are each equipped with capturing antibodies against exosomal markers. Upon exosome binding, shifts or intensity changes occur in the transmission spectral peaks related to the levels of the target markers. Using the nPLEX assay the protein expression of exosomes collected from the ascites of cancer and non‐cancer patients were profiled. The nPLEX assay was able to distinguish between exosomes from ovarian cancer patients with elevated protein expression of EpCAM and CD24 in comparison with non‐cancer patients (Im et al., 2014). The nPLEX assay showed sensitivities 10‐fold higher than western blot and chemiluminescence ELISA and was used to monitor the response to chemotherapy in ovarian cancer patients. Ascitic fluid was collected before and after chemotherapeutic treatment and the nPLEX assay used to show that patients responding to treatment had lower levels of exosomal EpCAM and CD24 or both compared to non‐responding patients (Im et al., 2014). In a further improvement of nPLEX, by using gold nanoholes rather than glass, increased sensitivity enabled multiplexed analysis of target surface and internal markers on single EVs (Min et al., 2020).

The QCM is a highly sensitive mass sensing acoustic biosensor. Either adhered or adsorbed to the surface of the sensor, changes in mass are detected in this piezoelectric device by changes in frequency of the gold‐coated quartz crystal and related by the Sauerbrey equation (Stratton et al., 2014). We previously used QCM with dissipation (QCM‐D) to monitor EV release from primary endothelial and fibroblast cells stimulated with agents affecting the cytoskeleton.

This was demonstrated as a real‐time loss in mass, giving an indication of the kinetics of EV biogenesis (2.4 × 10^6^ EVs released from 10^5^ prostate cancer cells over 1000s; 1.4 EVs per cell per minute). Similar estimates have been given by others (M. Bebelman et al., 2020; Verweij et al., 2018). The QCM has also been used to monitor EV release (from PC12 and NG108‐15 cells) after stimulation with increased potassium and by spontaneous endocytosis (Cans et al., 2001) or to study the absorption properties of red cell EVs (Románszki et al., 2020).

Another adaptation of this technology for EV research involves characterization of EV markers by QCM‐D. Although highly sensitive, compared to other methods, it is expensive and labor‐intensive requiring preparation of samples on assembled monolayers and of IgGs covalently immobilized on the sensor surface (Priglinger et al., 2021). With a view to making EV characterisation by QCM technology more accessible, therefore, (QCM coupled with impedance, QCM‐I) represents an improvement which was further linked to atomic force microscopy (AFM). This sensitive and label‐free immuno‐sensing method required minimal sample preparation, and enabled detection of mesenchymal stem cell (MSC) EVs via EV and MSC markers, the further use of AFM allowing the distinction of EVs and non‐vesicular particles (Priglinger et al., 2021).

An optofluidic smart phone‐based device called the mobile exosome detector (μMED) was developed by Ko et al. (2016) that can capture and analyze exosomes following mild traumatic brain injury using in vitro and murine models. The μMED is a handheld diagnostic device utilizing a smartphone camera for fluorometric data acquisition. The μMED device is based on a key innovation that uses both negative and positive selection of exosomes using immunocapture microbeads to isolate on‐chip. The positive and negative microbeads are incubated with serum for 30 min in an on‐chip compartment. The sample is then passed through the device using negative pressure which leads to the capture of the negative selection microbeads on a porous membrane and the remaining uncaptured exosomes pass through. Downstream, the sample interacts with positive selection microbeads on a porous membrane where the exosomes of interest are captured. The depletion of exosomes that are not derived from brain cells is achieved through negative selection. This leaves only brain‐derived exosomes for analysis through immunolabeling with anti‐CD81 tagged microbeads. The signal from the captured exosomes is amplified using HRP‐conjugated antibodies against glutamate receptor 2, a marker for mild traumatic brain injury. The intensity of the fluorescence signal is acquired on the smartphone device via the camera. The μMED device enables the isolation of brain‐derived exosomes from mice serum without the need for ultracentrifugation which is time consuming and requires large equipment. In addition, the μMED device was shown to be a cheap, quick, and portable instrument to detect mild traumatic brain injury in comparison to expensive conventional procedures such brain MRI. The authors suggest the μMED device can be used as a point of care test due to the wide availability of smartphones.

#### 5.2.3 Fluorescence‐based techniques for single vesicle analysis

A fluorescence‐based lab‐on‐a‐chip platform was described by Deschout et al. (2014) to accurately measure vesicle size and concentration in interstitial fluid from human breast cancer tumors. Deschout et al. produced a microfluidic device which incorporated light sheet illumination for fluorescence single‐particle tracking analysis (FSPT). FSPT was previously reported to measure the size range and concentration of vesicles that are fluorescently tagged in bodily fluids without vesicle isolation or purification. The innovative combination of light sheet illumination with FSPT allows for the reduction of background noise which is a well‐known problem due to limited contrast occurring because of unbound fluorescent dyes or out of focus particles in FSPT.

Another, fluorescence‐based technique recently developed called single EV analysis (SEA) allows for the multiplexed measurement of biomarkers on single EVs (K. Lee, Fraser, et al., 2018). The SEA technique operates first with the immobilization of EVs on a microfluidic device with subsequent lab‐on‐a‐chip immunostaining and imaging of the captured EVs. Immunostaining is accomplished with fluorescent antibodies against EV markers such as CD9, CD63, and CD81 or cancer markers, in this particular study from three glioblastoma cell lines overexpressing EGFRvIII, EGFR, or IDH1‐R132. Imaging of the immobilized EVs was performed in cycles for three different biomarkers in successive rounds by quenching the fluorochromes and then repeating the staining process for the other markers. The intensity of fluorescence was measured on single EVs generating data on total vesicle counts and protein composition. Using SEA, Lee et al. discovered variations in EV markers and tumor markers on EVs. For instance, EVs derived from the Gli36 cell line overexpressing EGFRvIII were observed to be more positive for CD9, a marker for EVs than was found on other cell lines. Moreover, EVs exhibited a difference in tumor marker expression from the three cell lines studied. As anticipated, the EVs from the Gli36 cell line overexpressing EGFRvIII had a significant subpopulation positive for EGFRvIII (67%) whereas the EVs derived from Gli36‐IDH1‐132H cell line had a small subpopulation positive for IDH1‐R132.

#### 5.2.4 Paper‐based platforms for EV analysis

Paper‐based platforms are being actively researched as cheap, quick, portable, and simple analytical tools in clinical settings and point‐of‐care testing (X. Chen et al., 2018). A paper‐based aptasensor utilizes luminescence resonance energy transfer (LRET) from the upconversion of nanoparticles to nanorods which enables the detection and quantification of EVs. LRET is a spectroscopic technique that measures the distance and changes in distance between a donor and acceptor attached to a protein (Zoghbi & Altenberg, 2018). LRET is based on the resonance energy transfer between two spectroscopic probes in close proximity due to the overlap of the emission spectrum of the donor and the absorption spectrum of the acceptor (Dolino et al., 2014). The paper‐based aptasensor was fabricated by dividing the DNA aptamer sequence of the CD63 protein into two sections. One section is immobilized on the paper while the other section is attached to gold nanorods and mixed with EVs for application onto the paper. The presence of EVs triggers the merging of the two sections of the aptamer with CD63 protein present on the particle surface to form a complex that reduces the distance between the nanoparticles and nanorods inducing luminescence resonance energy transfer. The green luminescence emitted by the nanoparticles is quenched and this change in the intensity of the luminescence emissions is captured by an imaging camera. The detection of EVs is calculated using the quenching rate and upconversion luminescence and the concentration of particles (X. Chen et al., 2018). This paper‐based aptasensor developed by Chen et al. was reported to be highly sensitive with a limit of detection of 1.1 × 10^3^ particles/μl and was performed in 30 min proving to be a suitable assay in clinical settings.

## 6 CONCLUSIONS AND FUTURE DIRECTIONS

This review has included classical methods that have been used by laboratories throughout the world to isolate, detect and characterize EVs. We also have critically analyzed some of the challenges that further development of EV technologies face. With a view to developing EV‐based liquid biopsy, the field still needs to overcome low yields and purity, high costs, overly complex procedures, and lack of standardization. Ideally EV isolation should not be a prerequisite for analysis and the field must strive for an integrated procedure that would be quicker, requiring fewer steps. Novel methodologies open up the possibility of detecting EVs from small clinical samples. The electrochemical biosensor systems such as iMEX quantifying EVs from μl samples of unprocessed plasma are a positive move promising liquid biopsy as a point‐of‐care in cancer diagnostics and in monitoring response to treatment. With EVs being found in bodily fluids, especially urine and saliva, the noninvasive nature of these methods means that EV‐based liquid biopsy holds amazing promise for personalized medicine, but all the models and protocols mentioned in this review will still need extensive preclinical experience to prove reliability.

Looking ahead, molecular cargoes of EVs must be more fully characterized, both in health and disease, as this will help us better understand the role EVs play in disease progression. One of the biggest challenges facing the field is assigning different biological functions according to EV subtype. Although some such differences of function have been ascribed to certain sEV and lEV populations, this knowledge will require better characterization of EV subtypes based on mode of biogenesis or specific composition. It is also likely that there will be tailored isolation/purification procedures according to what EVs are being used for, for example, whether for diagnostics or therapy. For use in therapy, EV isolation is likely to require more rigorous and large‐scale methodologies than are needed for diagnostics. Eventually, however, it is likely that integrated technological advances will arrive enabling detection of markers without prior EV isolation.

## AUTHOR CONTRIBUTIONS

**Karina De Sousa:** Conceptualization (lead); writing – original draft (lead); writing – review and editing (equal). **Izadora Rossi:** Writing – original draft (lead); writing – review and editing (equal). **Mahamed Abdullahi:** Writing – original draft (equal). **Marcel Ivan Ramirez:** Writing – review and editing (equal). **Dan Stratton:** Writing – review and editing (equal). **Jameel Malhador Inal:** Conceptualization (equal); supervision (equal); writing – original draft (equal); writing – review and editing (equal).

## CONFLICT OF INTEREST

The authors have declared no conflicts of interest for this article.

## RELATED WIREs ARTICLE

Progress in extracellular vesicle biology and their application in cancer medicine

## Data Availability

Data sharing is not applicable to this article as no new data were created or analyzed in this study.
